# Structural performance of a climbing cactus: making the most of softness

**DOI:** 10.1098/rsif.2021.0040

**Published:** 2021-05-12

**Authors:** Anil K. Bastola, Patricia Soffiatti, Marc Behl, Andreas Lendlein, Nick P. Rowe

**Affiliations:** ^1^ Institute of Active Polymers, Helmholtz-Zentrum Hereon, Kantstrasse 55, 14513 Teltow, Germany; ^2^ Department of Botany, Federal University of Parana State, Curitiba, Paraná, Brazil; ^3^ Institute of Chemistry, University of Potsdam, Karl-Liebknecht-Strasse 24-25, 14476 Potsdam, Germany; ^4^ AMAP, Univ Montpellier, CIRAD, CNRS, INRAE, IRD, Montpellier, France

**Keywords:** climbing cactus, succulence, biomechanics, rheology, skin–hydrogel–core structure

## Abstract

Climbing plants must reach supports and navigate gaps to colonize trees. This requires a structural organization ensuring the rigidity of so-called ‘searcher’ stems. Cacti have succulent stems adapted for water storage in dry habitats. We investigate how a climbing cactus *Selenicereus setaceus* develops its stem structure and succulent tissues for climbing. We applied a ‘wide scale’ approach combining field-based bending, tensile and swellability tests with fine-scale rheological, compression and anatomical analyses in laboratory conditions. Gap-spanning ‘searcher’ stems rely significantly on the soft cortex and outer skin of the stem for rigidity in bending (60–94%). A woody core contributes significantly to axial and radial compressive strength (80%). Rheological tests indicated that storage moduli were consistently higher than loss moduli indicating that the mucilaginous cortical tissue behaved like a viscoelastic solid with properties similar to physical or chemical hydrogels. Rheological and compression properties of the soft tissue changed from young to old stages. The hydrogel–skin composite is a multi-functional structure contributing to rigidity in searcher stems but also imparting compliance and benign failure in environmental situations when stems must fail. Soft tissue composites changing in function via changes in development and turgescence have a great potential for exploring candidate materials for technical applications.

## 1. Introduction

Cacti are well known for structural and physiological adaptations allowing their survival in hot and dry climates. Upright cacti are emblematic of water-stressed environments and are well known for their ribbed stems, tough outer skins, soft fleshy cortical tissue and inner ‘core’ of stiffer woody tissue [1]. Previous studies have greatly advanced knowledge on the structure and function of self-supporting cacti [2–4], but little is known about the structural organization and biomechanics of climbing cacti. Climbing plants need to adapt to quite different physical constraints compared to self-supporting plants. They need to reach across gaps to find supports and thus need to develop adequate stiffness and rigidity in young ‘searching’ stages of growth [5,6]. Most vines and lianas are also well known for developing highly flexible stems later in development to protect the slender climbing stems from failure since tree branches are constantly moving in the wind and for surviving tree falls and branch failures [7]. Little is known about how the ‘soft’ cactus organization, which is adapted for storing water with bulky soft tissues, might be adapted for the mechanical needs as a climbing plant.

**Figure 1.**
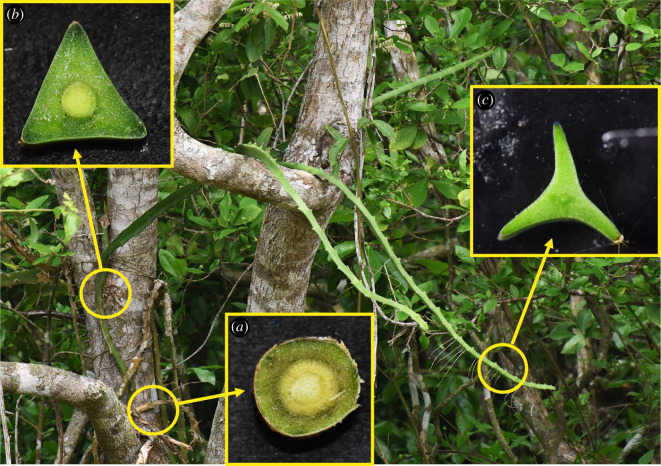
*Selenicereus setaceus* (Cactaceae). The species is a tree climber and develops three different stem shapes according to the phase of growth: basal older stems are circular in cross-section (*a*); the root climbing phase is triangular in cross-section (*b*); and young apical stems (searchers) have a winged profile (star-shaped) (*c*).

**Figure 2.**
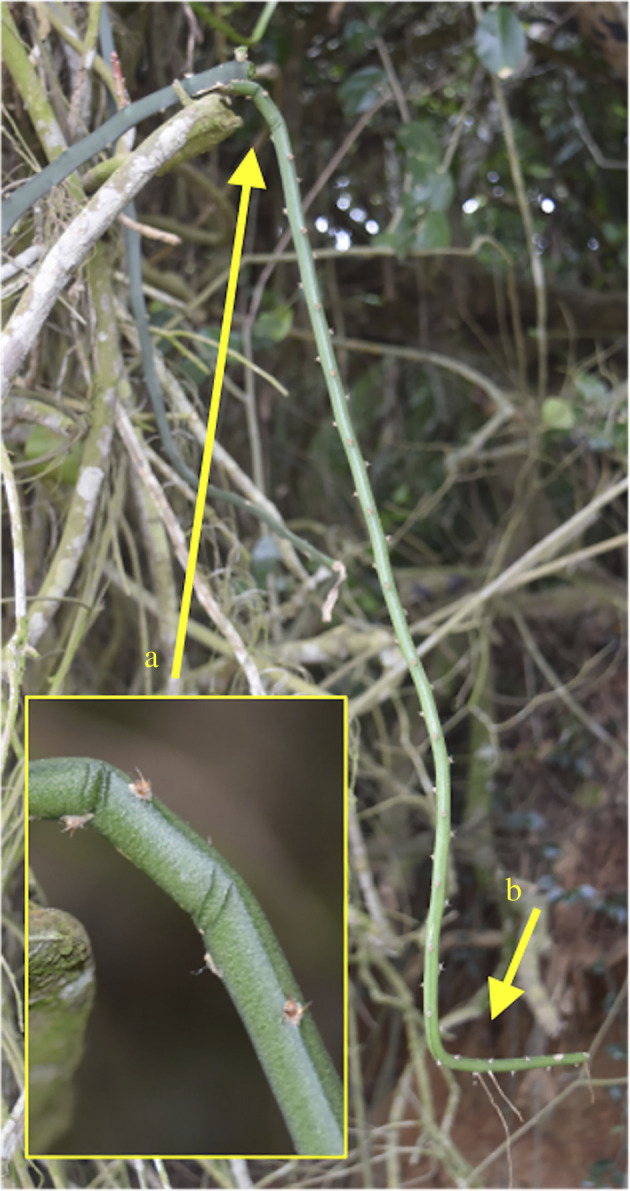
Failure in bending of the *Selenicereus setaceus* stem in understorey vegetation, Restinga forest, southern Brazil. A searcher has overreached its critical buckling length and/or been dislodged from a support. Although the base (arrow a) has buckled the apex is still growing and has renewed its searching behaviour (arrow b).

A recent study on the South American species *Selenicereus setaceus* (Cactaceae) has highlighted how changes in overall stem geometry and structural Young's modulus can optimize stem rigidity for a searching–climbing habit across diverse substrates [8]. These vary from highly ribbed apical ‘searcher stems’ that reach and locate supports to more basal triangular and circular cross-sectional organizations of the attached and climbing stems (figure 1). In the following study, we focus on the mechanical and swelling properties of individual tissues of *S. setaceus* and assess how such high levels of soft tissue can apparently maintain a climbing habit more usually seen in slender woody plants. We discuss how such relatively soft structures can nevertheless develop climbing forms that can search for supports and furthermore investigate how they might also be resistant to mechanical failure under real-world conditions where mechanical perturbation and failure is an ever-present risk for slender climbing stems (figure 2).

A fleshy cortex is present in many cacti and is composed of large thin-walled cells and scattered isolated mucilage cells or mucilage secreting canals, which produce mucilage [9]. Mucilage is a complex heteropolysaccharide formed by different sugars and is mainly involved in water storage. It has a high capacity to bind with water [10] and swells when exposed to water forming colloidal and viscous suspensions [11]. Mucilage is excreted into intercellular spaces of the cortex [9] acting as an apoplastic capacitor by retaining and passing water to surrounding cells thus regulating cell water content [12]. During dry periods, mucilage releases water, which is passively incorporated into parenchyma cells and maintains cell turgor [12]. This mechanism is probably necessary for mechanical support during young stages of development prior to the development of stiff lignified tissues. Since hydrogel-controlled turgor is important for maintaining tissue stiffness and stem rigidity during early growth, we suspect that its structural organization plays a key role in maintaining the ‘searching’ behaviour of young stems of the climbing cactus.

Changes in stem turgor and changes in cross-sectional shape and stem orientation, especially by hydrogel-mediated mechanisms, are of particular interest for bioinspired soft robotic applications that seek to provide actuation, control and modulation of physical properties in artificial systems [13–17]. Cacti have come under scrutiny recently as possible role models for bioinspired technologies [18]. Such model species are of particular interest since they use readily available materials in the surrounding environment with a minimum of energy expenditure.

We assess the functional roles of the tissues during development from searching for supports to attaching to diverse substrates as a climbing plant. We investigated macroscopic characteristics in the field with bending, tensile and swelling tests close to sites of growth and then carried out fine-scale studies on the rheology and compression properties of the stem under laboratory conditions. An important macroscopic feature is the ability of the stems to store water and thus swell and de-swell according to levels of hydration and turgor. We assessed to what extent stem segments and their tissue components were capable of swelling and de-swelling and to what extent the cactus was capable of morphing from star-shaped to circular cross-sectional shapes according to levels of swelling.

A key question concerns the need for young searchers to support their self-weight during the early self-supporting, searching phase of growth and also maintain their mechanical stability when faced with changes in environmental conditions such as wind and humidity/temperature. For this early stage, we measured the tensile properties of the outer skin, the bending stiffness (Young's modulus) and rigidity of the central woody tissue cylinder and its contribution to the rigidity of the whole stem. In this paper, we refer to mucilaginous tissue as cortical tissue that contains mucilage cells and in which abundant free mucilage exudes when the stem and cortical tissue is cut.

We then determined rheological properties of the mucilaginous tissue and compared transverse and longitudinal compression properties of the mucilaginous tissue and whole stem segments including wood cylinder tissue for all three stages of growth represented by searcher stems (highly lobed cross-section), climbing stems (triangular cross-sections) and older basal stems (rounded cross-sections) (figure 1) [8].

We hypothesized that compressive strength will likely differ between different developmental phases of the plant. Since the plant has different components (wooden core, mucilaginous tissue and skin) it is anticipated that the wooden core contributes more to rigidity and strength in older stages of growth. Moreover, since mucilaginous tissue comprises largely soft components with thin cell walls, large lumens, parenchyma and large quantities of both cellular and intercellular mucilage we anticipated that its mechanical properties might be similar to those of typical hydrogels (water-swollen polymers). If so, they likely influence stem shape and geometry according to the degree of hydration of the bulky cortex especially for younger stages of growth before secondary growth of the wood cylinder. In a previous paper, we focused on the overall life history and strategies of this climbing cactus as a potential role model for robotic artefacts capable of growth [8]. In this paper, we analyse in detail the component parts of this model species and explored the following questions.

What are the properties, organization and mechanical roles played by the outer skin?

What are the mechanical properties of the bulky cortex and its mucilaginous tissue, do they vary from the younger to the older part of the plant and are they comparable to properties of known hydrogels?

To what extent can stems change shape via swelling and de-swelling of the soft tissue components?

How much do soft mucilaginous tissues and the central woody core contribute to the rigidity of searcher stems of *S. setaceus* and to what extent does water availability influence swelling and de-swelling of the cortex, its mucilaginous tissue and overall stem shape and geometry?

## 2. Methods and material

### 2.1. Biological material

Segments of the cactus stem were collected from the Restinga coastal lowland dry forests in the region of the Armação dos Buzios town (22°44′49″ S, 41°52′54″ W, 174 km north of Rio de Janeiro city), Rio de Janeiro State. Sampling included 20 apical searcher, 20 climbing stem and 20 basal stem segments. Stem samples were cut with sharp secateurs from healthy plants showing little or no damage or disease.

### 2.2. Bending tests

Three-point bending tests [19] were carried out on 20 whole stem segments representing ‘searchers’ (star-shaped apical stems) using a portable Instron machine (In-Spec 2200, Instron Corporation, Norwood, MA, USA). After testing entire stem segments the wood cylinder was carefully removed with the aid of a scalpel and kept under moist conditions before being tested in three-point bending (electronic supplementary material, S1).

### 2.3. Tensile tests

Strips of cactus ‘skin’ were cut away with a scalpel from searcher stems tested previously in three-point bending. Strips of 7–15 mm in width and 1.5–3 mm in thickness were carefully removed and kept in moist conditions prior to the tests, keeping an initial length–width ratio greater than 10. The strips were clamped firmly in the upper and lower grips in order to avoid damage and slipping during the test. The samples were submitted to tensile tests using the portable Instron machine (electronic supplementary material, S2).

### 2.4. Stem anatomy

Following bending and tensile tests, stems were sectioned using a HM 650 V vibrating blade microtome with section thicknesses ranging from 20 to 200 µm. For further details on staining protocols and microscopy, see electronic supplementary material, S3.

### 2.5. Swellability tests

Swellability tests were performed on slices of the stem, 2–3 mm thick in cross-section for 20 rounded, 20 triangular and 20 star-shaped stems. Four adjacent slices were cut with a sharp razor from each stem. One complete section was submerged in a shallow dish containing demineralized water and the adjacent section placed in 0.5 M sucrose solution. For the remaining two slices, a triangular wedge was cut from each stem slice. The resulting notched stem and an isolated wedge of one slice were immersed in demineralized water and the cut segments of the other placed in 0.5 M sucrose solution (sucrose PA, Bioquimica Brazil). All stem slices were photographed immediately after being placed in the water or sucrose solution; they were left immersed for 6 h at room temperature after which they were re-photographed. A period of 6 h was chosen since pilot trials indicated that both maximum swelling and de-swelling occurred by 6 h submersion at room temperature. Changes in cross-sectional area of entire and notched stems and their main tissue areas were then calculated using the Image analysis software Optimas v. 6.5.172, Media Cybernetics, Inc., Rockville, MD, USA (electronic supplementary material, table S1).

### 2.6. Rheological measurements

All rheology and compression experiments described below were conducted within 10 days of sampling and stored in airtight containers at 4°C after arrival at the laboratory. In this condition, samples of the cactus stems have been found to stay fresh for more than a month (P. Soffiatti 2020, personal communication); stem segments were freshly prepared immediately before each experiment. Fresh slices of tissue, 8 mm diameter and 1.5 mm thick, were dissected from the three different stem segments (circular, triangular and star-shaped); three samples were tested from each segment (see electronic supplementary material for details on sampling and preparation, figure S4*a–h*).

Rheological experiments were performed on a MARS III rheometer (Thermo Scientific) (electronic supplementary material, S4). The minimum size of the rheometer plates is 8 mm in diameter. Samples were cut from strips of tissue dissected from the cactus stem to a thickness of 1.3–2 mm and were trimmed to a circular shape to fit the holder with a punch. Each sample was placed on the rheometer, after which measurements were performed at a constant temperature of 23°C. For an explanation of the different rheological tests, namely time sweep, amplitude sweep and frequency sweep, see electronic supplementary material, S4 and S5.

### 2.7. Compression tests

As a complement to the field bending tests, we studied contributions of stem components (wooden core, mucilaginous tissue and skin) to the compressive strength of the cactus stem. A 10 mm long stem segment was cut from each sample of the cactus in order to maintain a low length to breadth ratio to avoid instability and buckling during the test [20]. As a result, tests were undertaken at length to breadth ratios below 1.

The compression tests on mucilaginous tissue were performed on a rheometer (MARS III rheometer) and on a universal testing machine (Zwick/Roell Z1.0). Details are discussed in §3.5. The compression tests were continued at a compression rate of 2 mm min^−1^ and at a constant temperature of 23°C until the sample was deformed. Five samples were measured for each compression experiment (electronic supplementary material, figure S4*b–h*).

Samples were compressed in two directions: axial compression, where the direction of the force is along the length of the sample and radial compression in a direction normal to the axis length (electronic supplementary material, figure S4*e*). Medium grade (120) sandpaper was used to prevent slipping during the test (electronic supplementary material, figure S4*d*). Force/displacement data were obtained from compression tests as overall/effective stiffness. In addition, force at failure (N) and rigidity (N mm) were used to study the compressive behaviour of the plant using the force–displacement response.

### 2.8. Data handling and statistics

Sampling of stem segments aimed to extract branches from different plant individuals, but this could not be guaranteed because of the clonal life history of the plant. Representative circular, triangular and star-shaped branch segments can be accepted as different branches from mostly different individuals. All primary fieldwork and laboratory data and measurements are available at the figshare link. Statistical tests included non-parametric Kruskal–Wallace ANOVA followed by *post hoc* multiple comparisons tests to assess the significance of values between specific treatments. Non-parametric tests were chosen for field mechanical tests and swelling tests because of skewed and highly variable values particularly for rigidity, and variably positively skewed percentage datasets of swelling and de-swelling tests. Full datasets of field and swelling/de-swelling tests can be found at the figshare link. Parametric ANOVA were carried out on results from rheology and compression laboratory tests on the stem and mucilaginous tissue. Details of the tests can be found in the electronic supplementary material and full datasets on figshare. Each group sample (base, triangle and star) was drawn from a normally distributed population. Statistical tests were carried out with Microsoft Excel, Statistica (StatSoft, Inc., 2013) and using R-studio (v. 3.6.1; R Foundation for Statistical Computing, Vienna, Austria; www.r-project.org) (see electronic supplementary material, tables S2–S6)

## 3. Results

### 3.1. Bending properties of searcher stems

**Figure 3.**
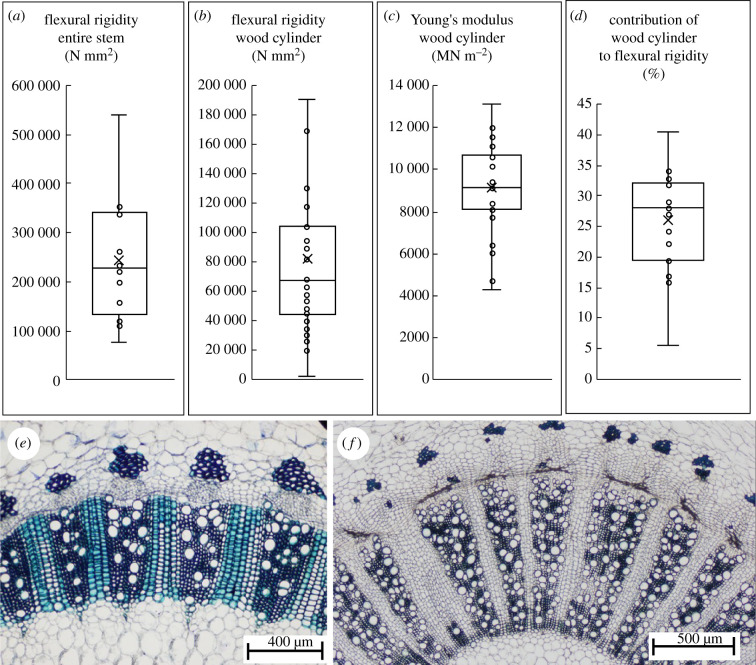
Wood stiffness and contribution to rigidity in searcher stems*.* (*a*) Flexural rigidity (*EI*) of the entire stem and (*b*) of the wood cylinder. (*c*) Wood Young's modulus (*E*). (*d*) Contribution of wood to the rigidity (boxplots: x = mean, line = median, 1st and 3rd quartiles, max and min). Transverse section of wood cylinder of (*e*) star-shaped stem and (*f*) circular stem.

The overall rigidity of stems varied by a magnitude of approximately five (figure 3*a*) across the size-range of searchers while the overall rigidity of wood varied by a factor of 10 (figure 3*b*). Young's modulus of woody tissue in star-shaped stems varied from 2900 to 19 000 MN m^−2^ (mean 9100 ± 2900 MN m^−2^) from younger to older developmental stages (figure 3*c*). This wide variation by a factor of three likely represents a gradient from very young wood cylinders to later more lignified stages of growth in searcher stems. Bending tests indicated that the central wood cylinder contributed to only 5.6–40.1% of the bending rigidity of the stem (mean 26.1 ± 8.29) (figure 3*d*). The narrow band of wood in searcher stems comprises axial regions of narrow thick-walled lignified fibres and vessels alternating with lignified ray tissue areas (figure 3*e*; see electronic supplementary material, S3). Searcher stems had very small wood cylinders with radial diameters varying from 0.08 to 0.54 mm (mean 0.28 ± 0.10). Older circular stems developed broader wood cylinders with early dense wood comprised of similar alternating vascular regions and rays as searcher shoots and then later wood development with alternating segments of vascular regions with less dense vessel-rich tissue alternating with unlignified ray tissue (figure 3*f*; see electronic supplementary material, S3d).

In summary, the wooden core contributes to only a limited amount of bending rigidity even over a wide range of searcher diameters. This means that bending rigidity of the gap-spanning, early searching phase must rely on the soft cortex–outer skin composite for 60–94% of stem rigidity.

### 3.2. Cactus skin and cortex

**Figure 4.**
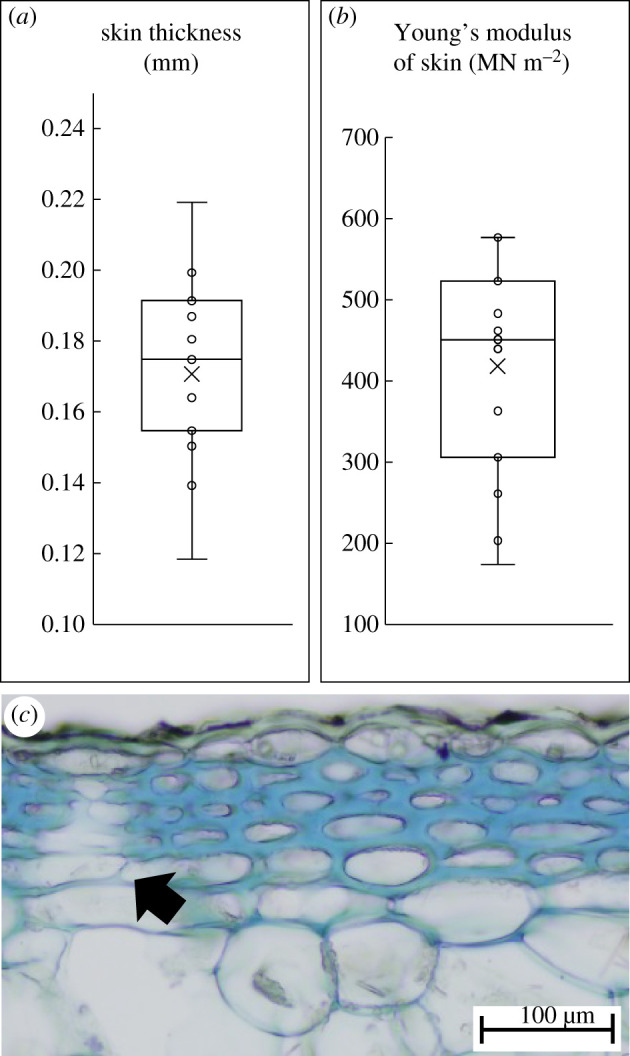
Properties of the outer skin of searcher stems. (*a*) Thickness of outer ‘skin’. (*b*) Young's modulus of skin in tension (boxplot parameters as in figure 3). (*c*) Transverse section of skin of star-shaped stem.

The skin of younger star-shaped and triangular stages of growth is composed of 4–6 layers of thick-walled collenchyma cells and an outer epidermis and cuticle. The collenchyma cells are axially elongated and overlap each other with pointed end walls. The bounding structure is very thin compared to the bulk of the mucilaginous tissue it surrounds and varies in thickness from 0.12 to 0.22 mm (mean 0.1707 ± 0.026 mm) (figure 4*a*). Young's modulus of the cactus ‘skin’ from the young star-shaped searcher stems showed values ranging from 174 to 577 MN m^−2^ (mean 418 ± 129 MN m^−2^) (figure 4*b*). The thick-walled collenchyma layer is interrupted by stomatal chambers which form open chambers in the mucilaginous tissue below (figure 4*c*, arrow).

**Figure 5.**
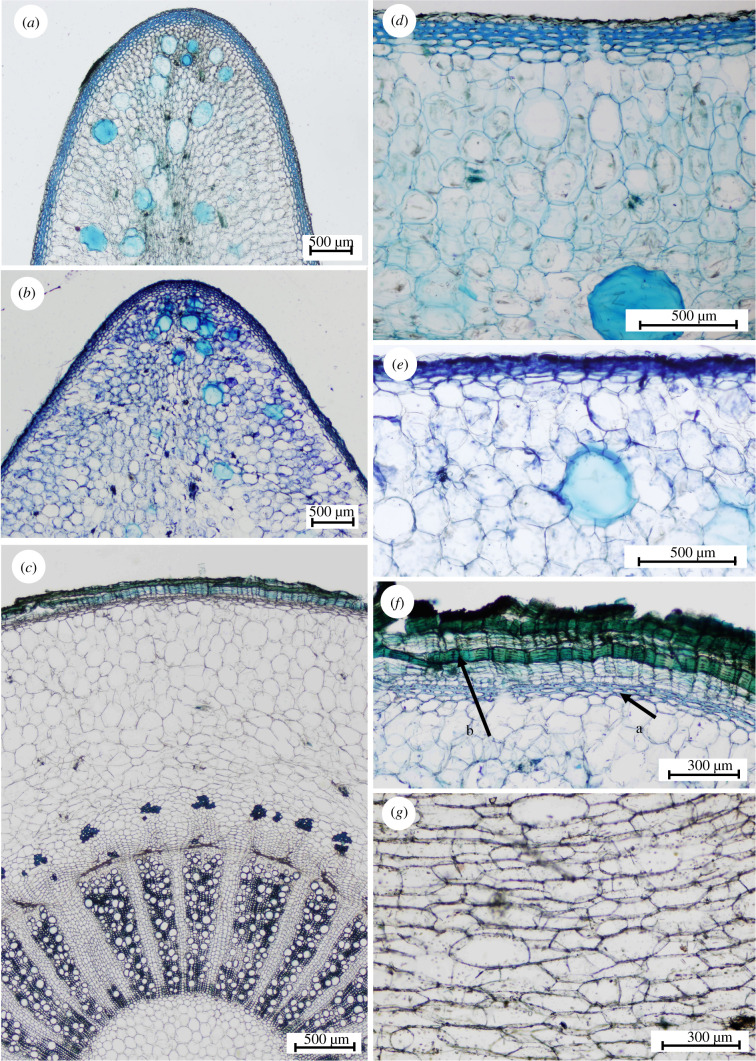
Internal organization. (*a*) Arm of the star-shaped stem with the cortex of large parenchyma cells and many mucilage cells. (*b*) Arm of the triangular stem with fewer mucilage cells. (*c*) Circular stem with bark tissue. (*d,e*) Outer skin and cortex of star-shaped (*d*) and triangular (*e*) stems. (*f*) Bark tissue of circular stem (arrow b) that replaces initial skin (arrow a) which is shrinking and fragmenting. (*g*) Cortex of circular stems with tangentially strained dividing cells.

The cortex of the young searchers is composed of many large mucilage cells in the lobed extensions of the stem (figure 5*a*,*d*). However, the cortex of the triangular stems has a more compact cellular organization with fewer, large-diameter mucilage cells (figure 5*b*,*e*). Older rounded stems have fewer mucilage cells (figure 5*c*,*f*) and show evidence of cortical disruption (figure 5*g*) with tangential straining and compensatory cellular proliferation ensuring the cortex remains intact during the expansion of the wooden core. Further anatomical changes in older rounded stems include the loss of the first-formed outer skin and its replacement by a periderm tissue formed by a phellem composed of lignified cells alternating with suberized cells (figure 5*f*; see electronic supplementary material, S3*e,f*) and cork parenchyma cells (phelloderm) (figure 5*f*).

### 3.3. Swell and de-swell tests

All stages of development and all treatments including entire, notched and wedge, showed significant levels of swelling and de-swelling of the cross-sectional area after submersion for 6 h in demineralized water or 0.5 M sucrose solution (electronic supplementary material, table S1).

**Figure 6.**
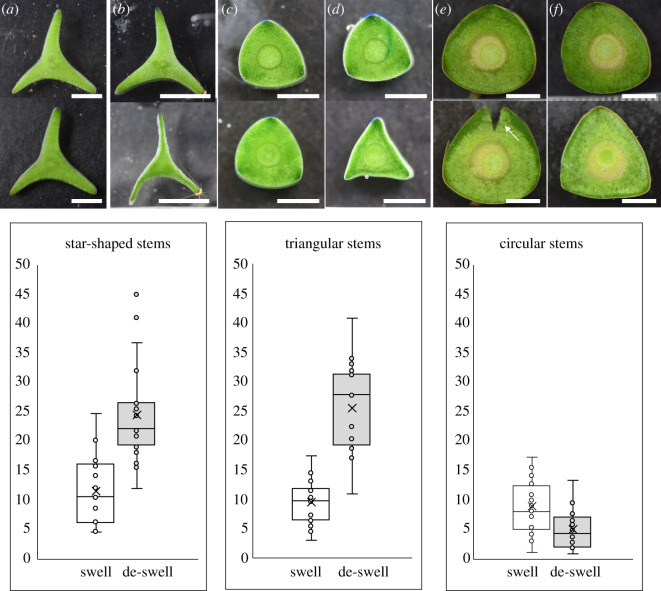
Swelling tests in demineralized water (*a*,*c*,*e*) and de-swelling tests in 0.5 M sucrose solution (*b*,*d*,*e*) on whole cross-sections. Scale bars = 5 mm. Percentage gain or loss in cross-sectional area after swelling and de-swelling tests for each stem type (boxplot parameters as in figure 3).

**Figure 7.**
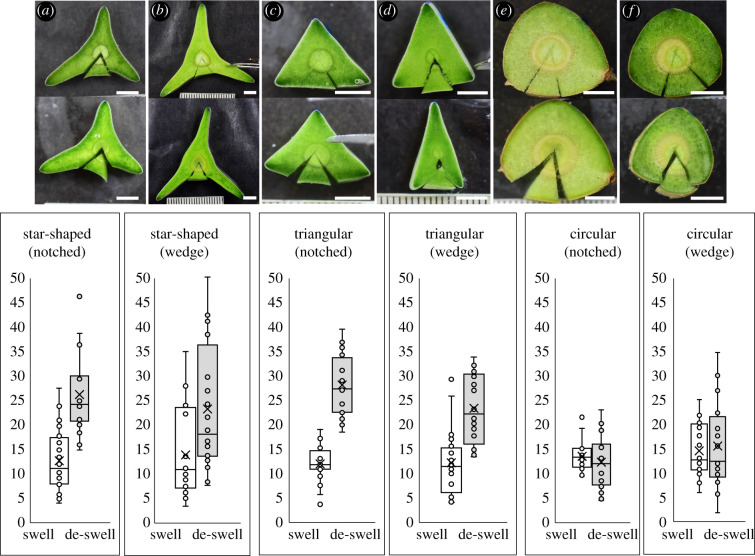
Swelling tests in demineralized water (*a*,*c*,*e*) and de-swelling tests in 0.5 M sucrose solution (*b*,*d*,*e*) on notched and wedge cross-sections. Scale bars = 5 mm. Percentage gain or loss in cross-sectional area after swelling and de-swelling tests for each stem type (boxplot parameters as in figure 3).

Whole stem sections showed 9–12% of swelling in water with no significant differences between star-shaped, triangular and circular stem segments (table 1, K-W, *H* = 2.07, *p* = 0.3544) (figure 6 and table 1). De-swelling of whole stems showed a different pattern. Star-shaped and triangular stems showed levels of de-swelling over double that observed for swelling, and values for circular stems showed significantly lower levels of de-swelling (table 1; K-W, *H* = 22.70, *p* ≤ 0.001) (figure 6 and table 1). Measurements of notched stems and the dissected wedges (figure 7) provided comparisons of swelling and de-swelling when the main tissue prone to swelling and de-swelling—the soft mucilaginous cortical tissue—is partly released from containment from the stiffer tissues of the outer skin and internal wood cylinder. Measurements indicated that the soft cortex was the principal tissue that changed in the cross-sectional area during the tests (table 1).

**Table 1.** Percentage gain or loss in cross-sectional area after swelling and de-swelling tests of growth stages prepared as whole stem, notched stem and wedged segments. (Kruskal–Wallace ANOVA by ranks, different letters indicate significant difference.)

sample	star	triangular	circular	*p*
swell				
whole stem	11.61 (±5.88)	9.45 (±3.64)	8.88 (±4.60)	*p* = 0.3544
notched stem	12.69 (±6.56)	11.96 (±3.78)	13.57 (±3.01)	*p* = 0.2991
wedge	13.64 (±9.16)	12.20 (±6.80)	14.60 (±5.30)	*p* = 0.3220
whole stem (% cortex)	12.85 (±6.19)	10.26 (±3.89)	10.18 (±5.52)	*p* = 0.4040
de-swell				
whole stem	24.52 (±8.46)^b^	25.53 (±7.47)^b^	5.03 (±3.27)^a^	*p* ≤ 0.001
notched stem	26.23 (±8.21)^b^	28.13 (±6.34)^b^	12.37 (±5.43)^a^	*p* ≤ 0.001
wedge	23.35 (±12.70)^a^	23.35 (±7.13)^b^	15.58 (±9.11)^a^	*p* = 0.009
whole stem (% cortex)	23.47 (±6.51)	28.78 (±9.46)	6.17 (±3.76)	*p* ≤ 0.001

Notched stems of star-shaped and triangular stems and their wedges showed little difference in the degree of swelling and de-swelling compared to entire segments (figures 6 and 7 and table 1). Circular stems, however, showed significant increases in swelling and de-swelling in both notched stems and isolated wedges compared with whole stem swelling (figure 7) beyond the 5% observed in whole stems to levels of 12% (notches) and 16% (wedges) (table 1). These increases in mechanically ‘freed’ segments did not, however, reach the relatively high levels of de-swelling observed in younger star-shaped and triangular stems. Swelling and de-swelling initiated changes in shape, especially in star-shaped and triangular stages. Rounded stems became more triangular, triangular sections became more winged and winged sections became more narrowly winged. Swelling of older circular stems resulted in the rupture of the outer skin and cortex in half of the stems tested (figure 6*e*, arrow) but not in younger stems.

In summary, younger stages of growth with none or very small amounts of wood (0–2% cross-sectional area) and a collenchyma skin can swell up to about 10% in water and this does not change much if the stem is cut open to reduce the confining effect of the outer skin and the wood cylinder. De-swelling of these stems is much more marked (about twice that of swelling) for entire stems that were collected in a well-hydrated native state from the field (figure 7 and table 1).

### 3.4. Rheological properties of the cortex

In order to study the dynamic properties of mucilaginous tissue, changes in storage modulus (G′) and loss modulus (G″) were measured by rheological tests in different conditions with respect to changes in time, stress amplitude, frequency and temperature (electronic supplementary material, figure S5*a–h* for definitions and details) (see Methods section for experiment definitions). G′ reflects the real part of the complex modulus of the deformation and is a measure for the restorability of the elastic deformation, whereas G″ reveals the imaginary part of the complex modulus and is a measure for the viscosity. It reflects the energy that is lost by inner friction into heat. In this way, the determination of G′ and G″ from the cacti enables one to determine the elastic deformation.

#### 3.4.1. Time sweep

**Figure 8.**
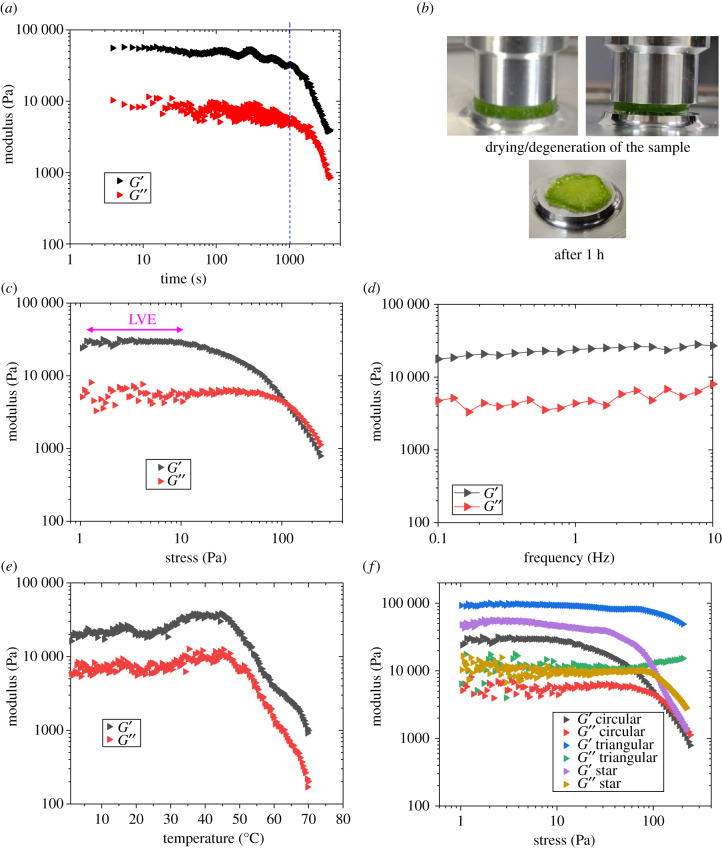
Rheology of mucilaginous tissue. (*a*) Storage and loss moduli/time. (*b*) Drying of the sample. (*c*) Amplitude sweep: storage and loss moduli/stress at 1 Hz. (*d*) Frequency sweep: storage and loss moduli/frequency. (*e*) Temperature sweep: storage and loss moduli/temperature. Results in (*c*–*e*) are for the circular section of the cactus (see electronic supplementary material, figure S5*a*–*h*, for data for other stages) Storage and loss moduli versus stress of the mucilaginous tissue for different sections (circular, triangular and star-shaped).

Time sweep measurements allowed us to understand the structural composition of tissues and their degeneration during shearing over time. An example of results after a time sweep is given in figure 8*a*. During the time sweep, the elastic portion of the curve (G′) is always higher than the viscous portion (G″) of the material. This indicates that the mucilaginous tissue is a viscoelastic solid. Secondly, both moduli (G′ and G″) are decreasing with increasing time, which implies changes in the structure of the mucilaginous tissue. It was found that the mucilaginous tissue sample started to dry over time (figure 8*b*). Therefore, the decrease in the moduli must be attributed to the shrinkage of the samples over time. When the sample starts to dry, the measuring plates lose their proper contact with the sample and the measurements become unreliable. As a result, an apparent decrease in moduli was observed. Similar observations were made with the samples from the star-shaped and triangular sections of the cactus (electronic supplementary material, figure S5*a,b*). On average, the moduli are apparently linear up to 1000 s (less than 15 min) as indicated by the blue dotted line. This first type of test (time sweep) provides the essential finding that the rheological experiments must be conducted before drying of the samples, which is within 15 min after the preparation of samples. Thus, all further experiments were conducted within 15 min after the preparation of the sample.

#### 3.4.2. Amplitude sweep

This test allowed us to understand the deformation behaviour of mucilaginous tissue in the non-destructive deformation range, widely known as the linear viscoelastic (LVE) region, and to determine the upper limit of this range. The result of the amplitude sweep at 1 Hz is given in figure 8*c* where the moduli start to decrease with increasing stress, indicating that the inner structure of the mucilaginous tissue starts to break in a brittle way. In the low-stress region, both moduli are seemingly constant. This region is known as the LVE region (figure 8*c*). A similar trend was observed for samples from star-shaped and triangular sections (electronic supplementary material, figure S5*c*,*d*). On average, if the stress generated within the materials is above 20 Pa, decreases in moduli are highly marked. Thus, the LVE region for the mucilaginous tissue is maintained up to approximately 20 Pa of stress.

#### 3.4.3. Frequency sweep

The time-dependent behaviour of the mucilaginous tissue in the non-destructive zone (i.e. the LVE region) was explored in frequency sweep experiments. High frequencies were used to simulate fast motion over short time-scales, whereas low frequencies simulated slow motion on long time-scales or at rest. The result of the frequency sweep (figure 8*d*) indicated that the mucilaginous tissue possesses seemingly constant moduli over the test frequency range. In other words, mucilaginous tissue does not change its properties (viscoelastic solid to viscoelastic liquid) over time. A similar trend was observed for mucilaginous tissue from the triangular and star-shaped sections (electronic supplementary material, figure S5*e,f*).

#### 3.4.4. Temperature sweep

The temperature-dependent behaviour of the material without chemical modification was investigated by temperature sweep experiments. Similarly, the temperature sweep can be performed to study the thermal behaviour of polymers (*T*_g_ and *T*_m_), thermal behaviour of crystallizing solutions and dispersions, as well as temperature-dependent behaviour with gel formation or curing (sol/gel transition *T*_sg_). The results of the temperature sweep are given in figure 8*e*. Moduli are seemingly linear below 45°C; however, they start to decrease considerably after 50°C. This can be attributed to the drying of the samples over time as already more than 15 min were needed to reach a temperature of 50°C. This result from the temperature sweep results also supports the results that were obtained in the time sweep. A similar observation has been made for the mucilaginous tissue from different sections of the cactus (electronic supplementary material, figure S5*g*,*h*). Nonetheless, it can be concluded that the mucilaginous tissue does not change its properties or phase over 0–45°C.

In summary, the mucilaginous tissue from the triangular sections of the plant possessed a higher modulus under rotary shear, which was investigated further under compressive loading. On average, the storage modulus of the mucilaginous tissue of the circular sections is about 30 kPa, that of the triangular sections is about 90 kPa and that of the star-shaped sections is about 50 kPa (figure 8*f*).

### 3.5. Compression test results

#### 3.5.1. Mucilaginous tissue compression

**Figure 9.**
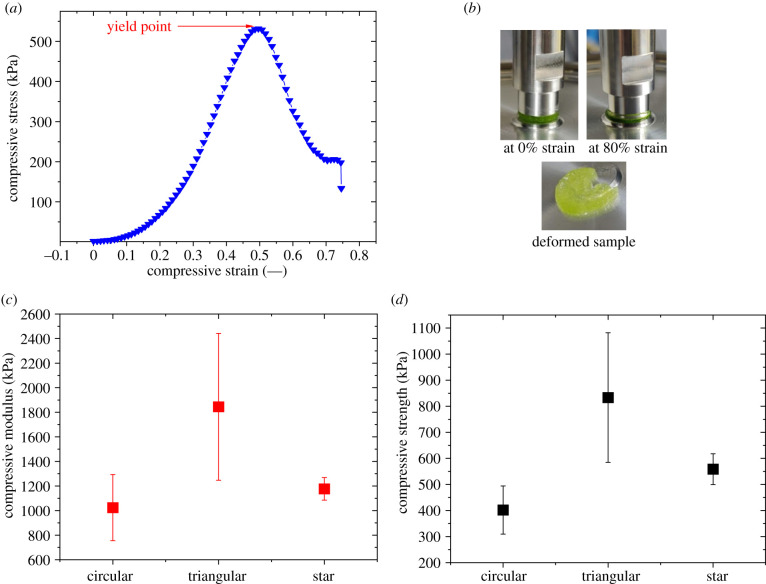
Compression tests of mucilaginous tissue. (*a*) Compressive stress/compressive strain. (*b*) Set up and effect of compression on the sample. (*c*) Compressive modulus and (*d*) compressive strength of mucilaginous tissue from different stages. (Means and standard deviations $\left(\bar{X}\pm \mu \right)$; see electronic supplementary material, figure S6, for details.)

The stress–strain curve of the mucilaginous tissue has a nonlinear response at a low strain level (less than 20%) and followed by a linear response and finally a yielding/failure at about 50–60% strain (figure 9*a*). This type of stress–strain response is very similar to typical representatives of physically or covalently cross-linked hydrogels such as alginates (physical) and polyacrylamide (covalent) [21,22]. As shown in §2.7 above, samples were found to be damaged or deformed after the test (figure 9*b*). The result presented in figure 9*a* is of the mucilaginous tissue from the circular section of the cactus. A similar type of stress–strain response was observed for the mucilaginous tissue from different sections of the plant (electronic supplementary material, figure S6). As described in §2.7, the compressive modulus (obtained from the slope of the stress–strain curve) and the compressive strength (maximum stress or stress at yield/failure) were also obtained for further comparisons and analysis.

Figure 9*c*,*d* shows the compression moduli and compressive strength of mucilaginous tissue from all three different sections (circular, triangular and star-shaped) of the cactus. Compressive properties (modulus and strength) of the mucilaginous tissue are higher for the triangular section of the cactus (*p* < 0.05) (electronic supplementary material, table S4) and softer in circular and star-shaped sections. These results are in line with those from the rheological measurements. The compressive strength of the mucilaginous tissue can vary from 300 to 1080 kPa and these values are similar to the typical compressive strength of a physical or chemical hydrogel [21,22]. It has been reported that the compressive strength of alginate-based hydrogels can be tuned from 100 to 700 kPa by changing the concentration of the Ca^2+^ ions, which are necessary to form the physical cross-links [21].

#### 3.5.2. Compression of entire stem segments

**Figure 10.**
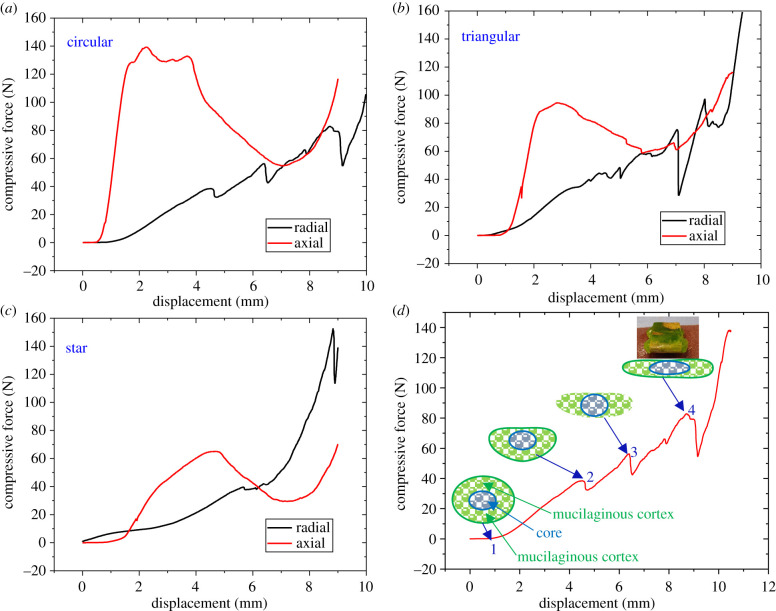
Compression force–displacement curves of different developmental stages of the cactus (*a–c*) based on the radial and axial orientations. (*d*) Failure events during compression of the circular stem during radial compression.

Because the developmental stages of the cactus differ widely in cross-sectional shape (circular, triangular and star) it was difficult to obtain comparable stress–strain responses. Therefore, the responses of different stem shapes were observed via force versus displacement curves (figure 10*a–c*). Overall, the responses of radial and axial compression were easily distinguishable with axial compression curves being consistently higher than those in radial compression (figure 10*a–c*). This can be attributed to the presence of the wooden core along the axis of the stem. The cactus stem can be described as a multimaterial system having a distinct architecture built up forming separate tissues rather than as a composite material with homogeneously distributed components [23]. In axial compression, the wooden core provides the resistance to compressive force while, in the radial compression, outer skin and mucilaginous tissue are responsible for the initial response. Secondly, the force–displacement responses are also different for different developmental stages of the cactus. Thirdly, the failure or breakage of the cactus stem can be identified by the abrupt drop in force marking the failure event of the plant structure. However, it must be noted that failure events are different in radial and axial compression. In axial compression, a clear yield point can be observed which indicates the failure of the wooden core. In radial compression, multiple yield points can be observed where yield points can be attributed to the failure of mucilaginous tissue and then the wooden core or the skin.

Multiple yield points are more notable for the circular section of the plant (figure 10*a–c*) and includes a series of well-defined events (figure 10*d*). Event 1 refers to the native state of the stem before the application of a load. Events 2 and 3 mark the failure of the mucilaginous tissue located below and above the wooden core. The third failure event (event 4) marks the failure of the inner wooden core. A similar type of failure event has been reported by Spatz *et al*. [24].

**Figure 11.**
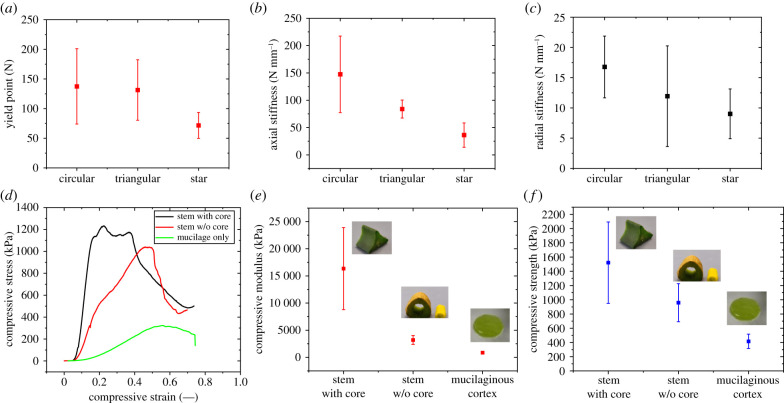
Compression properties of stems and core and with core removed. (*a*) Yield point, (*b*) axial stiffness and (*c*) radial stiffness. (*d*) Stress–strain response of cactus stem with core/without core. (*e*) Compression modulus and (*f*) compressive strength. Mean values with standard deviations $\left(\bar{X}\pm \mu \right)$.

Figure 11*a–c* provides a summary of maximum force at failure (in the axial direction) and axial and radial stiffness of the different sections of the cactus. A common trend can be observed where, in general, the older developmental stage (i.e. circular section) is always stronger or stiffer than the younger part (i.e. triangular section) followed by the younger part (i.e. star section). The axial stiffness of the circular section is 4 times higher (on average) than that of the star section. On the other hand, the radial stiffness of the circular section is only 1.7 times higher than that of the star section. ANOVA tests reveal that the difference in the compressive properties of the circular and triangular sections is not significant (*p* > 0.05). On the other hand, the difference in the compressive properties between circular or triangular and star section is significant (*p* < 0.05).

#### 3.5.3. Compression of stem without core

Comparisons of the stress–strain response of the stem with core, without core and mucilaginous tissue of the cactus show clear differences in the stress–strain response of different components (figure 11*d*). Firstly, as expected the yield point and the slope of the stress–strain curve are higher for the stem with core and lowest for the mucilaginous tissue. Secondly, the failure of the stem with core occurs at a low strain level while the failure of the mucilaginous tissue occurs at higher strain levels. Further comparison is provided by obtaining compressive modulus and compressive strength with the average values and standard deviation. Figure 11*e,f* provides a summary of the compressive properties of the cactus. It is found that the compressive modulus of the stem is 5.1 times (on average) lower if the wooden core is removed. The compressive modulus of the stem with core found to be 19 times (on average) higher than that of mucilaginous tissue. On average, the outer skin and mucilaginous tissue contribute to about 20% of the overall strength of the cactus.

## 4. Discussion

### 4.1. Searcher stem rigidity and structure

Climbing plants are known to develop stem rigidity in young ‘searcher’ stems to cross voids and reach supports for colonizing host vegetation [6]. Many woody vines and lianas produce a cylinder of stiff wood early in development or a thick layer of fibre tissue around the periphery of the stem [6]. The climbing cactus shows a different strategy by maintaining rigidity by enclosing a bulky but soft hydrogel-like tissue within a thin layer of hypodermal skin [8]. Our measurements of this indicate that its thickness and Young's modulus will not contribute significantly to stem rigidity directly as a stiffening element but acts as a bounding layer that contains swelling and de-swelling of soft cortical tissue essential for maintaining turgor driven rigidity. Our results indicate that the contribution to the rigidity of the wood cylinder in young searcher stems is relatively little (below 50%) and that the cactus searchers depend on the mechanical attributes of the outer skin and cortical tissue. Further, detailed analyses and modelling would be desirable to measure the role of the outer skin more precisely in maintaining the mechanical integrity of the stem while under different levels of water availability and turgor. The structure and organization of the collenchymatous hypodermis is similar to that of many other cacti in lacking lignification [9].

The overall mechanical architecture of *S. setaceus* shows a number of differences to the mechanical architectures previously identified in upright columnar and even creeping or procumbent cacti with a similar skin–cortex–central woody cylinder organization as well as rib-like extensions [3,4,25]. Those studies show that the skin plays an important role in stiffening especially in younger stages (apex) and that there is a correlation between the amount of wood fraction and the increase in stiffness of the stems, although its contribution to stiffness in bending is relatively low.

### 4.2. Soft tissues and developmental change

The changes in mechanical properties of the mucilaginous tissue observed are potentially explained by changes in its anatomical organization (figure 5*a–g*). These explain the rheological and compression experiments, where the storage modulus of mucilaginous tissue was smaller for the younger star-shaped sections and oldest circular sections, but greatest for intermediate-aged triangular stems. Interestingly, different responses of mucilaginous tissue observed for different growth stages were retained in different types of rheology tests (time sweep, amplitude sweep, frequency sweep and temperature sweep). The fact that the mucilaginous tissues include both cell walls and mucilage, compared to synthetic materials such as hydrogels which lack cellular contents, potentially explains why the storage modulus of the cactus mucilaginous tissue was found to be higher than that of typical hydrogels, which display storage moduli less than 10 kPa [26,27]. Further analyses on the biological cactus tissue and its individual components will be needed to verify this.

Younger stages of growth maintain relatively high levels of rigidity via turgor before the development of a woody core. Turgor is a fundamental driving force that provides strength to plant tissues and organs, and contributes decisively to growth and movement [28]. Mucilaginous tissue in these stages is ‘less soft’ than that of older circular stems where there has been a change in properties after the development of the resistant core. This change was also suggested in swelling tests where the capacity to swell fully was probably confined by the development of secondary tissues in the outer (skin) and inner (woody core) of the stem structure. In summary, the wooden core is providing more and more strength to the cactus and the mucilaginous tissue plays a diminishing role for mechanical support. Values of Young's modulus for xylem cylinders show relatively high values compared with measurements of modulus based on other species of cactus [3,4,9,25].

The cellular ‘skeleton’ of mucilaginous tissue appears to be structurally different between growth stages and probably linked to the initial need to ensure turgor stiffness in young searchers and the later need for compensatory cellular proliferation to keep pace with the growth of the internal wood cylinder. Further studies on the cellular organization would shed light on how fine structural modifications of the low mass skeleton material can fine tune and optimize the rheological and compression behaviour of natural and potentially artificial hydrogel systems.

### 4.3. Role of the outer skin and mucilaginous tissue

The outer skin of young stages (figure 5*a*,*b*,*d*,*e*) differs in function from the outer peridermal and decaying old collenchyma skin of older stages (figure 5*c,f*). Observations of the outer ‘skins’ of older stems indicated that indeed the structure of the skin shows a fragmentation of the previous collenchyma and forms a later bark tissue (periderm) (figure 5*f*). The young skin can mechanically withstand high turgor pressures driven by the hydrogel-like mucilaginous tissue and thus ensure rigidity of searchers via the pressure generated by cells of the cortex under turgor, as also observed in other studies with other species of columnar and creeping cacti [4,25].

The swell and de-swell tests showed that whole sections of star- and triangular-shaped stems can show high levels of swelling and de-swelling as well as overall changes in cross-sectional shape. In older circular stems, however, the swell and de-swell tests showed less swelling and de-swelling and, furthermore, half of the specimens tested actually split apart as a result of high turgor of the cortex (figure 6*e*, arrow). Increased development of the wooden core and compression and proliferation of the mucilaginous cortex were accompanied by the disintegration of the original elastic skin and its replacement by a phellem (bark-like tissue) all of which imply a marked shift in mechanical architecture. These older stages of growth, therefore, rely less on a hydrostatic skeleton for mechanical support. Interestingly the outer bark-layer of older circular stems can keep pace with the ‘slow’ volume increase and slow growth resulting from the cellular proliferation of the wood and cortex, but swell experiments of entire stems showed that they cannot adjust to large ‘fast’ increases in volume resulting from rapid increases in turgor by the mucilaginous tissue. Although not tested in this study, this result suggests that the later formed peridermal skin is more brittle than the early formed hypodermis of younger stems.

### 4.4. Success and failure of a skin–hydrogel–core architecture

As a porous structure, the mucilaginous cortical tissue displayed a clear failure point under compressive loading, typically at a strain level of about 60%. As discussed above, compressive properties of the mucilaginous tissue also appear to be influenced by the stage of growth. In the rheological study, the mucilaginous tissue was found to be a brittle viscoelastic solid where measurements showed that if stresses generated within the mucilaginous tissue reached levels of about 20 Pa, the internal structures of mucilaginous tissue would start to deform. This inherent ‘limit’ for the compression resistance of the soft cortex would seem to have important repercussions on the safety limits and risks of especially young star-shaped and triangular-shaped stems in the real world for crossing long gaps, being dislodged from their support and being mechanically deflected by wind and rain. For example, if wind action (an important factor in the Restinga forest in Brazil) on searcher branches of the cactus was sufficient to generate 20 Pa of stress within the mucilaginous tissue, its structure would begin to break down and thus both the outer skin and wooden core would be exposed to higher loads and risk of failure. Similarly, for star-shaped searchers, if the self-weight of a branch were enough to generate 20 Pa of stress, again, the mucilaginous tissue would fail, and the mismatch in stress between wooden core and mucilaginous tissue would likely lead to excessive bending of the cactus stem and eventual mechanical failure.

As expected, the overall compressive properties (yield point, radial and axial stiffness) as well as bending rigidity and Young's modulus [8] of the plant are higher for older circular sections. On the other hand, even though star-shaped searcher stems optimize rigidity via shape [8], the compressive strength is always much less than in older stages of growth. Thus, at first sight, these searchers are probably susceptible to destructive failure more than other sections of the cactus by environmental perturbation such as wind or self-weight.

Interestingly, the failure of star-shaped searchers by local buckling near the base is relatively common in natural conditions (figure 2, arrow a). Stems fail after local buckling on the compression side of the stem with one or more folded areas on the lower surface. This kind of failure is linked to stages of growth where the cortical tissue becomes weakened by compression and the central core has not developed sufficiently to support the searcher. Despite the mechanical failure, in these natural situations, stems do appear to remain alive and even continue growth and exploring by axial growth at the apex but form a new position in the vegetation (figure 2, arrow b). It appears that even in these ‘worst-case scenarios' where the skin–mucilaginous tissue–core structure fails, the compliance of the soft tissue and the skin appear to protect the stem from complete breaks or open fracture surfaces.

## 5. Conclusion

Soft mucilaginous tissue is a key component of this climbing cactus and its life in dry habitats. The soft tissue can change properties during development from young searcher stems to older basal stages of growth. The mechanical architecture of the climbing cactus can be understood in a simplified way as a three-component skin–hydrogel–core organization. All three components change in properties and organization between different stages of growth. The main shift in overall development involves a change from reliance for mechanical support of a hydrogel supported turgor-skin organization that maintains rigidity for searcher stems to older stages with increasing wood tissue and less reliance on mechanical support from the cortex and skin. The absence of significant clusters of bands of lignified fibre tissue in the outer cortex of young searcher stems—a feature of great importance in many other climbing plants—means that stem rigidity relies on geometrical optimization (shape) and turgor for rigidity.

This architecture involves different kinds of tissue and different kinds of growth during the different functional needs of the plant. Changes in stem geometry via ‘fast’ swelling or de-swelling involve large volumes of the soft tissue of the hydrogel-like cortex according to water availability. On the longer term, ‘slower’ changes in geometry—such as increase in the wooden core for wider girth and stronger stems—require longer term, additive growth by cellular proliferation via meristems and new allocation of biomass.

The combination of ‘slower’ additive growth processes combined with ‘faster’ turgor driven swell and de-swell abilities is a highly adaptive architectural mechanism for plants [28]. This combination of different developmental features promises to be an attractive model for conceptualizing and transferring plant growth processes to new innovative materials and structures. Such combinations of developmental processes are widespread in the plant world and probably underpin a vast range of habits and adaptive movements, particularly among climbing plants. Climbing plants are well known for their flexibility, movements and sensitivity to external stimuli; especially for attaching to supports. Combinations of stiff and soft tissues that can adapt by growth or swelling and de-swelling are, therefore, attractive models for actuation and adaptive movements [15] of robotic artefacts but also as actuators for specific roles such as sensitive attachment mechanisms [16]. Furthermore, the specialized climbing succulent cactus shows also how the adaptive development of soft tissue in relation to other stem components can ensure rigidity of young stems that can also guard against catastrophic brittle failure, when the physical demands of the real world become unavoidable.

## Data accessibility

Additional information and data are available in electronic supplementary material, S1–S8. Raw data are available at the assigned figshare link.
